# The Work of Hospital Almoners—III

**Published:** 1917-07-28

**Authors:** 


### THE WORK OF HOSPITAL ALMONERS.—III.

### Address by Miss Cummins at the London School of Economics.

IN-PATIENTS.

THE almoner's work is now not confined to out-patients, for it was found after a time that exactly the same need for social work existed for in-patients, and in several hospitals assistant almoners for in-patients have been appointed. The patient is seen on admission, to secure: (1) That he or she is suitable for free in-patient treatment. (2) That admission to the hospital will not entail home troubles that can be avoided. Thus, if the patient is the wage-earner, help may be needed to keep the family, or, if the mother of the family needs admission, possibly her baby may have to be boarded out and a woman found to look after the home. (3) That patients who are in a position to do so can have an opportunity of arranging to contribute towards the upkeep of the hospital. Then the in-patient almoner is needed in the ward if new developments arise in the homes, or if convalescent aid or surgical instruments are required, or again very often the patients must be followed back to their homes and helped during the difficult period that often elapses before they are fit to resume their ordinary life. Or again, as you know, a certain proportion of patients must leave the wards with no prospect of ever resuming their normal life again, incurable. Some may have a few months to live, others may have years, and someone is needed to help rearrange things if unnecessary suffering is to be avoided, and the standard of life of a whole family is to be maintained. Perhaps four cases which I had the other afternoon will best illustrate this work.

I. The surgeon sent round about Mrs. C. He wanted her to be admitted that very day, and she had refused because of her baby and home. I found that her husband would not be back till ten o'clock, and besides the baby she had left two children with a neighbour. Well, the baby had to be sent to its grandmother for the night with a promise that I would move it to a babies' home next day. The husband was visited at his work and matters explained, and he agreed that he would manage for the night if he could find a decent woman to look after the children. Mrs. C. was so ill that she agreed to any feasible plan, and was cheered by the promise of daily reports on the children.

II. Next I was asked to see a man who was restless and uncomfortable in the ward. Sister said that he was worrying as to how his wife was managing, and he had to be seen and arrangements made for someone to visit his home next day.

III. Then the house surgeon came in to say that he wanted to give a pound for the case of a baby in the children's ward whom he greatly liked. He was sure that its parents did not give it enough food, but would I advise? Well, I happened to know that the parents were quite in a position to provide the necessary milk, etc., and that as a matter of fact we were already trying to educate the mother. It took a little time to satisfy him, and I promised to let him know if any difficulty should arise later.

IV. Then one of the sisters came down to ask if I could see a girl up in her ward who had been brought in a month ago, having taken poison. It was a miserably sad story, and sister thought that she wanted much more help than the month's convalescence that had been recommended.

But I will not weary you with more cases. Of necessity the work is much less simple than it was when it first started. Still the outline is much the same, and above all I would like to impress upon you the human side of it. The humour and tragedy of life are very constantly before us, and all day long and every day the figures in our foreground are constantly shifting and changing. First, it may be a single girl who has learnt from the doctor that she is pregnant, and whose life for the next few months will be constantly in touch with us. Then follows a small boy who, owing to bad language or disobedience, has been sent home from a convalescent home. Then follows a house physician, who wants milk for a baby in the district; and then a man who has to lie up for a time, and who wants help for his family to tide them over the difficult days. You will say that the almoner cannot have time for really getting to know the patients, and in a way you may be right, but it is extraordinary how often the intercourse does develop into real friendship. The hospital holds an extraordinary place in the hearts of the people, and its atmosphere is one in which friendship and affection kindle wonderfully quickly; so that, in spite of the constant flow to and fro, there is a residue that seems more or less always with us. I am sometimes asked how much time is devoted to each patient. It is impossible to say. A few minutes may be all that is needed to satisfy one about one patient, while the next may need three-quarters of an hour's hard concentration, and that only as an introduction to many subsequent interviews.

ORGANISATION.

But perhaps I have not laid enough stress on one side of the almoner's life, which nevertheless is an important one. Her activities are truly chiefly employed in bringing all her force to bear on the patients with whom she comes in contact, but, in addition to this, in a big hospital she has a great deal of organisation thrown upon her which is intimately connected with her work.

If you consider the work of a big out-patients' department, with its casualty department, its special departments, and its district work, and realise that the same patient is often passed from one to the other, and perhaps later on to the wards, you will see how the information and statistics which are of vital interest must be codified and passed from hand to hand, so that information, once obtained, may be of use to doctor, nurse, and visitor concerned with the case during the whole course of treatment.

The fact that the almoner is the centre of information and inquiry about all matters relating to the general social condition of a patient means that the daily number of inquiries about, and appointments for, special cases is a big one; and to lighten successfully the labours of the medical staff and to ease the condition of the patient attending, one needs a capable organiser, who can balance and arrange for the work to be carried through with the least amount of friction possible.

Then, again, a good almoner should be an authority on the questions relating to municipal authorities, the Poor Law, the War Pensions Statutory Committees, the Insurance Committees, and so forth. The doctors con-

**(Concluded on p. 347.)**

☛ NOTE.—The previous articles appeared on July 14, p. 299, and July 21, p. 319.

### THE WORK OF HOSPITAL ALMONERS.—III. (concluded from p. 342).

stantly come into contact with these, and the almoner should be available for information on any difficult points.

There is, in addition, the call for someone who can, when working in such close co-operation with nurse, patient, and philanthropic societies, individually sympathise, and recognise the danger of duplication in work done, and from the hospital, the centre of treatment, be able to join in or propose a scheme for carrying through the work with the least possible expenditure of time and labour.

TRAINING.

The work of an almoner implies very considerable interference with the lives of others. How can anyone dare to intervene in so important a matter without being well qualified to do so? The almoner, then, must receive from her training principles upon which to work. These principles will have to be applied with a basis of tact and intelligence in her dealings with the hospital patients. She will learn that the greatest asset in a man's troubles is the man himself; that, if help be needed, it should be given adequately, and so as to prevent, as far as possible, a recurrence of the trouble; that illness is only one incident in a long chain of events in a man's life; that hospitals, like other charities, have their limitations, and that these limitations must be recognised. Apart from this, it is clear that she needs a great deal of purely technical knowledge; it is necessary for her to understand the powers and duties of the public authorities, the Guardians, the medical officers of health, and so on. She must know what charities there are in the place in which she works, what they are prepared to do, and on what terms they will do it. She undergoes a course of lectures in economics and sociology in connection with this school, and is under the supervision of a tutor who directs her reading and discusses difficult points. During this period, which lasts about a year, she has also opportunities for practical work and study in the homes of the poor.

Then the candidate is sent to a qualified hospital almoner and learns the actual details of hospital work. This part of the training also lasts nine months. During that time the candidate works in the out-patients' department, and learns something of (1) the hospital life and etiquette, (2) how to deal with patients who are in difficulties, and (3) how to invite all forms of help, medical, municipal, and voluntary. (4) She learns to treat all diagnoses as strictly confidential. (5) She also learns to work with the doctors, and arranges that the patients who are sent up purely for consultative purposes do not drift into regular *habitués* of the hospital. During this part of her training the candidates have courses of lectures on physiology, hygiene, and home sanitation.

The most necessary qualifications are tact, judgment, force, imagination, and a certain sense of proportion. The almoner is only one small, though I believe very necessary, part of a large scheme for medical charity, and she will need to exercise much care and consideration in dealing with all the other parts if the greatest good of the patient is to be achieved.

The training is full of interest, and my experience has been that it has always been valued. An almoner needs to have good health. The tax on one's strength, both physical and mental, is great. The hours are long, and the demands for help ever increasing. Sometimes only a saving sense of humour helps one through the day. But I can heartily assure you that the profession of a hospital almoner is one full of human interest, and an almoner has the satisfaction of knowing that, however imperfect her own efforts, the work is worth while.

The selection and training of almoners is in the hands of the Hospital Almoners' Council at Denison House, Vauxhall Bridge Road, and all inquiries should be sent to the Secretary of that Council.

